# Concurrent trajectories of residential region in relation to a sustainable working life among Swedish twins

**DOI:** 10.1093/eurpub/ckad053

**Published:** 2023-04-08

**Authors:** Annina Ropponen, Mo Wang, Iman Alaie, Jurgita Narusyte, Pia Svedberg

**Affiliations:** Division of Insurance Medicine, Department of Clinical Neuroscience, Karolinska Institutet, Stockholm, Sweden; Finnish Institute of Occupational Health, Työterveyslaitos, Finland; Division of Insurance Medicine, Department of Clinical Neuroscience, Karolinska Institutet, Stockholm, Sweden; Division of Insurance Medicine, Department of Clinical Neuroscience, Karolinska Institutet, Stockholm, Sweden; Department of Medical Sciences, Uppsala University, Uppsala, Sweden; Division of Insurance Medicine, Department of Clinical Neuroscience, Karolinska Institutet, Stockholm, Sweden; Division of Insurance Medicine, Department of Clinical Neuroscience, Karolinska Institutet, Stockholm, Sweden

## Abstract

**Background:**

Residential regions may impact the possibilities to achieve a sustainable working life (SWL, i.e. not having interruptions due to sickness absence, disability pension or unemployment) due to disparities in social security and labour market. We aimed to investigate concurrent trajectories of regions and SWL among Swedish twins.

**Methods:**

National register data were used for the degree of SWL in each year, old-age pension, emigration, death and residential regions classified in three categories (cities; towns and suburbs; or rural areas) of Swedish twins in 1998–2016 (*n* = 80 398). Group-based multi-trajectory modelling and multinomial regression for relative risks with 95% confidence intervals were calculated.

**Results:**

The six-group solution had the best fit to data with trajectories: stable living in towns and suburbs with SWL (33.8%); stable living in cities with SWL (22.1%); stable living in towns and suburbs with increasing SWL (13.9%); stable living in towns and suburbs with lack of SWL (13.2%); stable living towns and suburbs with decreasing SWL (8.8%); and stable living towns and suburbs with decreasing and ultimately lack of SWL (8.3%). Age and being woman increased and being married and higher education decreased the likelihood of belonging to groups 2–6 (vs. 1).

**Conclusions:**

The simultaneous assessment of trajectories of three residential regions and SWL indicated that most people in Sweden seem to live continuously over time in towns and suburbs, but the degree of SWL may vary. More fine-grained assessment of residential regions would be needed to clarify the associations with SWL.

## Introduction

In the European Union, Sustainable Development Strategy^1^ holds and calls for longer working lives for the purpose of promoting equal sustainable working life at both ends of the working life as well as in the middle of the working career. Hence, this also calls for identification of characteristics and circumstances supporting sustainable work across the lifespan, both at the individual level, regarding the actual work conducted, and the work environment at large. Other important aspects are the macroeconomic and political conditions, with previously documented impact on sustainable working life.^2–4^ Differences between countries regarding the role and functioning of social security are evident. For example, long-term sickness absence (SA) is more prevalent in Sweden than in Finland, while on the other hand, Finland has higher rates of unemployment.^5^ However, similar disparities might appear even within a country due to differences in living areas. Although previous studies have addressed spatial and geographical differences in SA and disability pension (DP),^6–9^ studies are lacking for residential regions in relation to sustainable working life (i.e. not having interruptions due to SA/DP or unemployment) to the best of our knowledge.

Residential regions provide a measure for spatial differences as working and living conditions in different regions may vary depending on, for example local demographics, occupational composition, local attitudes, differences in education, disparities in transport facilities, socioeconomic strata, availability of jobs and disparities in healthcare system.^10–13^ On the other hand, working life is usually characterized by changes in employment as well as living and working conditions. That is, employment status may vary^14^ or there might be interruptions in working life due to SA, DP or unemployment, while this changes may occur independently or interlinked.^14^^,^^15^ Changes in sustainable working life may relate to regional characteristics considering that the risk of SA/DP has been found to be higher among people living in rural or semi-rural municipalities compared to urban municipalities or towns.^10^^,^^11^^,^^13^^,^^16^^,^^17^ Furthermore, sociodemographic characteristics (such as age and sex) in residential areas^10^^,^^17^^,^^18^ but also higher share of manual jobs, higher level of self-reported mechanical, physical and chemical exposure of the residential region^16^ can play a role for sustainable working life. However, to the best of our knowledge, previous studies have not addressed simultaneous changes of both residential regions and sustainable working life over an extended period.

Genetically informative samples such as twins might provide further understanding of the associations between residential regions and sustainable working life. This assumption is based on the known role of genetics in SA/DP^19^^,^^20^ but also on other research demonstrating genetic influences on socioeconomic circumstances more generally.^21^^,^^22^ Accounting for genetic similarity between twins in the associations between concurrent changes in residential regions and sustainable working life would add to the earlier knowledge based on other populations of unrelated individuals. Altogether, knowledge of simultaneous changes of residential regions and sustainable working life would be needed to target social security, occupational health care and support for employment in more efficient way.

In this study, we aimed to investigate concurrent trajectories of residential regions and sustainable working life among Swedish twins and assessed the genetic similarity between twins.

## Methods

The Swedish Twin project Of Disability pension and Sickness absence (STODS) includes the twins identified in the Swedish Twin Registry (STR) who were born between 1925 and 1990, i.e. 119 907 twin individuals. In this study, we utilized the STODS data that include national register data from the Swedish Social Insurance Agency for years 1994–2016 about SA/DP. Furthermore, we use the linkage to the Longitudinal integrated database for health insurance and labour market studies (LISA) Statistics Sweden (SCB) for 1994–2016 for unemployment^23^ and data on residential regions, classified into three groups according to Swedish municipalities for the degree of urbanization^24^ (cities that are densely populated areas; towns and suburbs as intermediate density areas; or rural areas which are thinly populated areas). Since data on residential regions were available comprehensively and reliable since 1998, we limited the follow-up from 1998 to 2016. This follow-up enabled us to estimate if the residential region for each twin individual remained the same or changed between years. Degree of sustainable working life was estimated through main labour market status in each year of follow-up using the definition: SA/DP (> 180 days with SA or DP benefits from the Social Insurance Agency); unemployment (> 180 days with unemployment benefits); old-age pension (more than half of yearly income from old-age pension); or employment (i.e. in paid work and did not fulfil the criteria SA/DP, unemployment or old-age pension). Emigration (from LISA) and death from Cause of Death Register from National Board of Health and Welfare were assessed for censoring. Hence, the final sample with complete data on residential regions, sustainable working life and factors of interest included 80 398 twin individuals.

Factors of interest at baseline in 1998 were genetic similarity based on monozygotic (MZ), dizygotic (DZ) same-sex and opposite-sex twins with also twins without known zygosity, age as calculated from baseline year and birth date and sex from STR and from LISA educational level [elementary school (≤9 years)/missing; high school (10–12 years); university/collage (>12 years)] and marital status (married; not married). Since occupational sector (private/public) based on LISA data for every year was assumed to play role both for residential regions and sustainable working life that was accounted as time-varying covariate in the statistical analyses as we could use the information of all follow-up years.

### Statistical analysis

First, we calculated descriptive statistics for means with standard deviations (SD) and frequencies with percentages. Then group-based multi-trajectory analysis was used^25^ to identify trajectories of residential regions and sustainable working life. Residential regions were treated as a categorical variable and sustainable working life as a dichotomous variable. Group-based multi-trajectory modelling is a form of finite mixture modelling to distinguish and describe subpopulations (clusters) existing within the studied population.^25^ A zero-inflated Poisson model and logit model of group-based multi-trajectory analysis with linear distribution were used. The goodness of model fit was judged by running the procedure several times with the number of trajectory clusters starting from one up to as long as the model converged. The goodness of fit was confirmed using the most parsimonious criteria of Bayesian Information Criterion, Akaike Information Criterion and average posterior probability of cluster membership. In addition to these, the smallest group size for trajectory groups was set *a priori* to 5%. Then, we conducted multinomial regression analyses to evaluate the relative influence of baseline sociodemographic factors and genetic similarity for the identified clusters. All analyses were performed with Stata/MP Statistical Software: Release 17 (StataCorp, College Station, TX, USA). The additional Stata module ‘traj’ was required to conduct group-based trajectory analysis.^25^ Since we used occupational sectors as time-varying covariate, we also ran trajectory models without such adjustment as a sensitivity analysis (see Supplementary data).

The study was approved by the Regional Ethical Review Board in Stockholm (2007/524-31; 2010/1346-32-5; 2017/128-32).

## Results

The mean age in the final sample was 41.6 years and the proportion of men and women was equal. Half of the study participants had an educational level of 10–12 years and 43% were married or living with someone. Also, the occupational sectors were equally distributed (Table 1).

**Table 1:** Descriptive characteristics of the study sample (*n* = 80 398)

In 1998	Mean	SD
Age (years)	41.6	13.2
	*n*	%
Sex		
Men	39 966	50
Women	40 432	50
Zygosity		
MZ	18 954	24
DZ opposite-sex	10 158	13
DZ same sex	23 562	29
Not known	27 723	34
Civil status		
Married	34 624	43
Not married	45 774	57
Education		
0–9 years	22 501	28
10–12 years	39 007	49
>12 years	18 890	23
Occupational sector		
Private	39 822	50
Public	37 489	47
Other/non-specific	3087	4

The six-group solution was the best based on the goodness-of-fit statistics (Table 2).

**Table 2:** Goodness-of-fit statistics of group-based trajectory analysis models

	Smallest group		BIC	AIC	APP
	*n*	%			
2-Cluster model	27 392	25	−2 349 405.85	−2 349 335.13	0.96
3-Cluster model	19 819	18	−2 306 632.11	−2 306 522.76	0.94
4-Cluster model	17 686	17	−2 238 704.28	−2 238 594.92	0.89
5-Cluster model	12 318	12	−2 217 296.74	−2 217 110.21	0.84
**6-Cluster model** ^a^	**8373**	**8**	**−2** **205** **884.88**	**−2** **205** **659.75**	**0.86**
7-Cluster model	8373	–	−2 205 929.47	−2 205 665.75	0.87

a The model presented is shown in bold.

AIC: Akaike Information Criterion; APP: average posterior probability; BIC: Bayesian Information Criterion.

The trajectory groups (Figure 1) were named and presented in the order of size as follows:

G3 (33.8%) Stable living in towns and suburbs with stable sustainable working life;

G4 (22.1%) Stable living in cities with stable sustainable working life;

G2 (13.9%) Stable living in towns and suburbs with increasing sustainable working life;

G6 (13.2%) Stable living in towns and suburbs with lack of sustainable working life;

G1 (8.8%) Stable living towns and suburbs with decreasing sustainable working life; and

G5 (8.3%) Stable living towns and suburbs with decreasing and ultimately lack of sustainable working life.

The regression results (Table 3) for trajectory group membership indicated that age was universally associated with increased likelihood of belonging to all trajectory groups in comparison to the largest group (3). Also, being women predicted increased likelihood and being married or having higher education were associated with less likely of belonging to these trajectories. The role of genetic similarity was mainly not statistically significant with few exceptions suggesting that familial confounding cannot be ruled out.

**Table 3:** Relative risk ratios (RR) with 95% confidence intervals (CI) for sociodemographic factors in relation to concurrent trajectory groups, residential regions and sustainable working life trajectory groups (trajectory group 3: stable living in towns and suburbs with sustainable working life as reference)

	G1		G2		G4		G5		G6	
	RR	95% CI	RR	95% CI	RR	95% CI	RR	95% CI	RR	95% CI
Age at baseline	**1.06**	**1.06, 1.06**	**1.02**	**1.01, 1.02**	1.00	1.00, 1.01	**1.09**	**1.09, 1.09**	**1.13**	**1.13, 1.13**
Sex (women)	**1.12**	**1.06, 1.18**	**1.32**	**1.24, 1.40**	**1.13**	**1.08, 1.18**	**1.20**	**1.13, 1.27**	**1.32**	**1.25, 1.38**
Zygosity										
MZ	1	Ref	1	Ref	1	Ref	1	Ref	1	Ref
DZ same sex	1.04	0.96, 1.09	0.96	0.88, 1.05	0.99	0.93, 1.05	**0.92**	**0.85, 0.99**	1.01	0.94, 1.09
DZ opposite sex	0.99	0.89, 1.11	**1.33**	**1.20, 1.47**	**1.10**	**1.01, 1.19**	**1.34**	**1.20, 1.50**	**2.30**	**2.08, 2.54**
Not known	1.01	0.94, 1.09	0.98	0.90, 1.06	0.96	0.91, 1.02	1.01	0.94, 1.09	**1.16**	**1.08, 1.25**
Marital status (married)	1.03	0.97, 1.09	**0.56**	**0.52, 0.59**	**0.73**	**0.70, 0.77**	**0.69**	**0.65, 0.73**	**0.40**	**0.38, 0.42**
Education										
0–9 years	1	Ref	1	Ref	1	Ref	1	Ref	1	Ref
10–12 years	**0.30**	**0.12, 0.71**	**0.37**	**0.18, 0.74**	**0.33**	**0.18, 0.59**	**0.13**	**0.06, 0.27**	**0.02**	**0.01, 0.03**
>12 years	**0.25**	**0.10, 0.60**	**0.21**	**0.10, 0.41**	**0.35**	**0.19, 0.64**	**0.09**	**0.05, 0.20**	**0.01**	**0.01, 0.02**

Statistically significant RR with 95%CI indicated with boldface.

Furthermore, although we adjusted for time-varying occupational sectors, we also tested their relative role for trajectory group memberships (data not shown). Working in the public sector at baseline was associated either with increased or decreased likelihood of these trajectories suggesting that the role of occupational sectors should be considered while assessing residential regions and sustainable working life.

## Discussion

This prospective study of over 80 000 Swedish twin individuals aimed to investigate concurrent trajectories of residential regions and sustainable working life. Six trajectory groups were identified, and the trajectory group with stable living in towns and suburbs with sustainable working life was the largest (34%). Other trajectory groups were smaller and indicated both stable living either in towns and suburbs or in cities with stable sustainable working life but also decrease and even lack of sustainable working life for those in towns and suburbs. These add to the previous knowledge based on studies of the differences in risk of SA/DP among people living rural or semi-rural municipalities compared to urban municipalities or towns.^10^^,^^11^^,^^13^^,^^16^^,^^17^ Since we also assessed sociodemographic characteristics (such as age and sex) and genetic similarity for the trajectory group memberships of residential areas and sustainable working life, our findings extend the findings from earlier research.^10^^,^^17^^,^^18^ However, although prior knowledge of simultaneous changes of both residential regions and sustainable working life is rare, there might be even more need for such studies as we included rather crude measure of residential regions, i.e. only three categories for the degree of urbanization^24^ (cities that are densely populated areas; towns and suburbs as intermediate density areas; or rural areas which are thinly populated areas).

Although our measure of residential regions indicated rather stable living area especially in towns and suburbs and in cities, we observed changes in sustainable working life. These findings could indicate lack of regional differences for sustainable working life since all measured categories were associated with stable and decreasing sustainable working life. Hence, this may also indicate that such differences may be more clearly indicated with more fine-grained levels of residential regions, yet to be confirmed in future studies. Prior research, focusing on SA/DP, has also utilized rather similar categorizations as in this study, i.e. rural or semi-rural municipalities and urban municipalities or towns.^10^^,^^11^^,^^13^^,^^16^^,^^17^ Although a trajectory analysis can be interpreted as descriptive, our concurrent trajectories reflect simultaneous changes of residential regions and sustainable working life. Such results provide knowledge for those responsible of social security, occupational health and even for efforts to reduce unemployment and should potentially be considered on more detailed levels in regions as we found no major differences between crude levels of regions.

In line with the assumption that sociodemographic characteristics (age and sex)^10^^,^^17^^,^^18^ and the assumption that residential regions might differ for their share of manual jobs, higher level of self-reported mechanical, physical and chemical exposure of the residential region,^16^ we tested these. We found that older age and being woman were associated with increased risk of belonging to all trajectory groups compared to the largest trajectory group. Instead, higher education (as a proxy for occupational status) predicted lesser likelihood for these. One can speculate that more fine-grained regions might be needed to further understand these associations. However, from a Swedish societal perspective, this seems to tell that residential areas with less likely providing possibility to sustainable working life should merit further investigation along with assessment of other influential factors such as disparities or the role and functioning of social security.

In this study, the use of twins enabled us to assess the role of genetic similarity for the trajectory group memberships. Despite the large sample size and determined zygosity, we did not find any effect of genetic similarity. However, studies with more sophisticated methods to evaluate the genetic influences would be merited to shed further light on this. Using twins in this large sample can be considered as a strength of this study as they add to the knowledge based on unrelated individuals. Another strength is the use of comprehensive register data that is free from recall and reporting biases and has no loss to follow-up. Also, the data on sociodemographic factors, residential regions and sustainable working life were of good quality since we assessed these factors using register data across rather long time, 19 years. Yet, since we used occupational sectors as time-varying covariate, that may have captured some variation due to employment circumstances. However, no study is without weaknesses. As speculated already, we used a relatively crude measure of residential regions including only three categories of the degree of urbanization. However, this categorization is internationally used, hence providing possibilities for further comparisons.^24^ Another limitation might be associated with the fact that we had limited number of influential factors to be assessed for investigation on whether those play a role in the group memberships. For example, health status, occupational group, local demographics or attitudes, disparities in transport facilities or disparities in healthcare system not available for the present study could be accounted in future studies.^10–13^ Moreover, our measure of sustainable working life did not include short (<180 days) interruptions due to SA or unemployment. That may play role especially among young individuals but also imply that at least for long-term interruptions our results might hold. However, if this measure should be re-evaluated, that could be addressed. Last, we need to acknowledge the fact that our data were Swedish with a well-developed welfare system. Hence, our findings may apply less to other countries without well-developed welfare systems than, e.g. other Nordic countries with similar welfare systems and societal wealth.

To conclude, our results with simultaneous assessment of trajectories of three residential regions and sustainable working life indicate that most people in Sweden seem to continuously live in towns and suburbs but the degree of sustainable working life may vary. Also, the one-fifth of people continuously living in cities has relatively stable sustainable working life. More fine-grained assessment of residential regions would be needed to clarify the potential differences in terms of sustainable working life.

## Supplementary Material

ckad053_Supplementary_Data

## Data Availability

The data that support the findings of this study are available from the original sources: the Swedish Twin Registry, Statistics Sweden, Swedish Social Insurance Agency and the Swedish National Board of Health and Welfare. Restrictions apply to the availability of the data used in this study based on the Swedish Twin project Of Disability pension and Sickness absence (STODS), which were used with ethical permission for the current study and therefore are not publicly available. Until now, studies have been rare on residential regions in relation to sustainable working life (i.e. not having interruptions due to SA, DP or unemployment). During working life, changes may occur either due to living and working conditions, but their linkage to residential regions and potential changes would provide understanding on their complex interplay and influential factors. As assessed with crude measure of residential regions, i.e. only three categories for the degree of urbanization, stable living was identified across 19 years of follow-up while sustainable working life was either stable or changed. Age and being woman increased while higher education or being married decreased the likelihood of belonging to the identified concurrent trajectories of residential regions and sustainable working life. For public health, assessment of residential regions should be more detailed to understand the interplay with sustainable working life. Concurrent trajectories of residential regions and sustainable working life with time-varying occupational sectors
