# Supplementary material for: Concurrent trajectories of residential region in relation to a sustainable working life among Swedish twins
Source: Eur J Public Health. 2023 Apr 8;33(4):596–600. doi: 10.1093/eurpub/ckad053 (PMC10393480; doi:10.1093/eurpub/ckad053)
Supplement: ckad053_Supplementary_Data [file ckad053_supplementary_data.docx]

**Supplemental Table S1** Goodness of fit statistics of group-based trajectory analysis models

|  | **Smallest group** | | **BIC** | **AIC** | **APP** |
| --- | --- | --- | --- | --- | --- |
|  | **N** | **%** |  |  |  |
| 2-cluster model | 29028 | 32 | -3587889.86 | -3587831.41 | 0.94 |
| 3-cluster model | 24921 | 23 | -2716417.92 | -2716327.00 | 0.96 |
| 4-cluster model | 15845 | 16 | -2682602.45 | -2682479.05 | 0.90 |
| 5-cluster model | 6925 | 7 | -2662512.53 | -2662356.6 | 0.92 |
| **6-cluster model*** | **6166** | **6** | **-2642699.40** | **-2642511.05** | **0.92** |
| 7-cluster model | 6166 | 6 | -2642736.88 | -2642516.05 | 0.92 |

* The model presented is shown in bold. BIC = Bayesian Information Criterion, AIC = Akaike Information Criterion, and APP = average posterior probability.

**
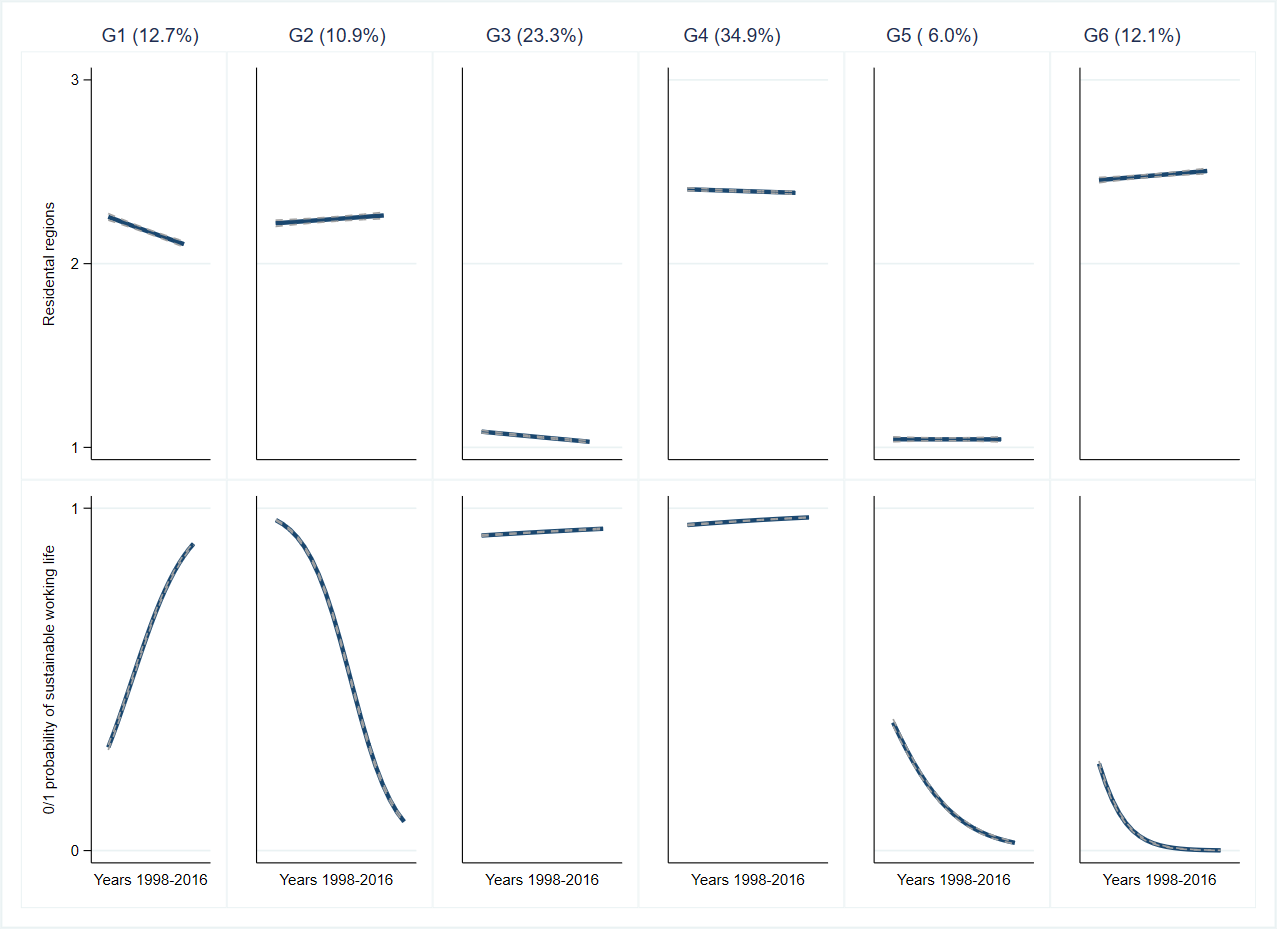
**

**Supplemental Figure S1** Concurrent trajectories of residential regions and sustainable working life
